# The Effects of Switching Non-Spatial Attention During Conversational Turn Taking

**DOI:** 10.1038/s41598-019-44560-1

**Published:** 2019-05-30

**Authors:** Gaven Lin, Simon Carlile

**Affiliations:** School of Medical Sciences and The Bosch Institute, University of Sydney, Sydney, New South Wales, Australia

**Keywords:** Auditory system, Attention, Perception, Human behaviour

## Abstract

This study examined the effect of a change in target voice on word recall during a multi-talker conversation. Two experiments were conducted using matrix sentences to assess the cost of a single endogenous switch in non-spatial attention. Performance in a yes-no recognition task was significantly worse when a target voice changed compared to when it remained the same after a turn-taking gap. We observed a decrease in target hit rate and sensitivity, and an increase in masker confusion errors following a change in voice. These results highlight the cognitive demands of not only engaging attention on a new talker, but also of disengaging attention from a previous target voice. This shows that exposure to a voice can have a biasing effect on attention that persists well after a turn-taking gap. A second experiment showed that there was no change in switching performance using different talker combinations. This demonstrates that switching costs were consistent and did not depend on the degree of acoustic differences in target voice characteristics.

## Introduction

Our external auditory environment is in a state of constant change. Sounds emerge, evolve, and shift in frequency and space across time. The ability to detect and adapt to these changes is central to how we interact with the world around us. A prime example of this is seen in the act of following a conversation in a cocktail party. In this dynamic environment, the focus of attention constantly shifts between multiple talkers as they take turns communicating. Keeping track of these auditory sources as the conversation ebbs and flows is crucial to understanding and participating in social exchanges.

Cocktail party environments are often difficult to navigate as they pose a perceptual challenge to the auditory system^1^. Firstly, listeners must separate the talker of interest from a sea of competing talkers and background noise. This feat can be achieved by directing top-down endogenous attention^2^, which has been posited to be object based^3,4^. Listeners can deploy their ‘spotlight of attention’ using features such as the voice or the spatial location to enhance the processing of a given target talker^5–8^. These acoustic consistencies allow the auditory system to stream and build a representation of sources across time^9–11^.

When the features of an auditory scene change however, the system must adapt and re-calibrate to new target features. Previous behavioural studies have shown that this shift in attention comes at a perceptual cost. For instance, listeners perform worse in multi-talker recall tasks when a target changes voice or location^9^. There is an increase in response time and processing errors when listeners have to switch attention between talkers^12–14^. With conversational stimuli, this equates to fewer words recalled, more masker confusions, and poorer comprehension especially after a turn-taking gap^15^.

These findings have been attributed to a disruption to streaming^9,16^, consequently leading to target re-orientation^6,17^ and increases in cognitive load and working memory demands^15,18^. Recent physiological studies have supported such a view, revealing markers of increased effort with switching attention using electroencephalography^13^ and pupillometry^19^.

While there has been considerable research into the mechanisms of attention switching, it remains to be seen how these limitations impact real world listening. Our previous work using conversational stimuli established the cost of switching attention in the spatial domain^15^. The current study aims to extend this by investigating the consequences of switching *non-spatial* attention during a conversation using multi-talker matrix sentences^20^. Here non-spatial attention refers to attention allocated to voice characteristics. In a cocktail party, unique talkers can be separated based on voice using features such as fundamental frequency, pitch, intonation and prosody^5^. All these distinguishing features can be used to stream a talker of interest independently of spatial cues.

Previous studies have shown that voice consistency between trials^17,21^ and within trials^9,22^ can enhance target intelligibility in multi-talker mixtures. Samson and Johnsrude^21^ showed, using co-ordinate response measure (CRM) speech^23^, that a target voice provides the auditory system with a template which guides listeners in extracting a repeating talker. Similarly, Bressler and Colleagues^22^ showed using digits embedded in time reversed speech, that this biasing/enhancement of a consistent voice happens automatically without awareness or intention. This phenomenon has also been seen in several voice priming experiments. The recognition of a target word masked by speech is significantly improved if it is preceded by a priming sentence spoken by the same talker^24–26^. Together, these studies highlight the benefit of voice continuity in informational unmasking and streaming of speech.

The current study expands on the literature by examining how these findings translate to a conversational task which not only differs in the complexity of the stimuli but also in the working memory demands. Multi-talker matrix sentences are used here to emulate the energetic and informational masking, voice changes, and multi element recall typically encountered in real world listening. This study used a novel two talker selective listening task coupled with a working memory probe task to assess the demands of non-spatial switching. Conversations can often be unpredictable with talkers pausing, resuming, or other talkers interrupting during turn-taking gaps. This study focused on uncued listening driven by endogenous tracking of speech based on a target name. Unlike previous priming tasks which have only examined the recall of single items^24–26^, this study examined the recall of two sentences before and after a turn taking gap to monitor streaming effects over the course of an unfolding conversation.

The first experiment aimed to establish the perceptual cost of a change in target voice. We expected enhanced performance with a repeating voice due to repetition priming effects^21,24–26^ and a reduction in the accuracy of word recall and increase in errors following a voice switch due to disruption to streaming^9,22^. In addition, we aimed to further isolate which component of streaming was disrupted by looking at the process of disengagement vs re-engagement of attention around a turn taking gap.

The second experiment in this study aimed to investigate the effect of voice differences in switching. Previous studies have shown that larger differences in voice characteristics can benefit auditory segregation^5,27,28^. But less is known of how different voices interact across a switch in attention. The current experiment investigated whether the cost of switching varied depending on the talker combinations used. Here different talkers were used to examine within-gender and between-gender voice switches.

## Methods

### Participants

Fifteen native English speakers (12 males, aged 21–30, mean = 27.1) participated in two auditory attention switching experiments. All subjects had normal hearing as assessed by a pure tone-audiogram (<20 dB hearing loss at frequencies between 250–8000 Hz), normal or corrected to normal vision, and no reported attention or cognitive deficits. All subjects gave written informed consent. This study was approved by the Human Research Ethics Committee, University of Sydney. All methods were performed in accordance with the relevant guidelines and regulations.

### General setup

Both experiments used speech material obtained from the Australian Matrix Sentence Corpus^20^. This corpus contains recordings of fixed syntax sentences comprised of interchangeable name, verb, number, adjective, and noun elements. Elements could be sampled without replacement from a pool of 5 × 10 possible words. Words were all 500 ms in duration with the exception of nouns, which were time stretched to 600 ms using Adobe Audition 3.0. This was done to mimic the prosodic lengthening of speech at phrase boundaries^29^.

**Figure 1:**
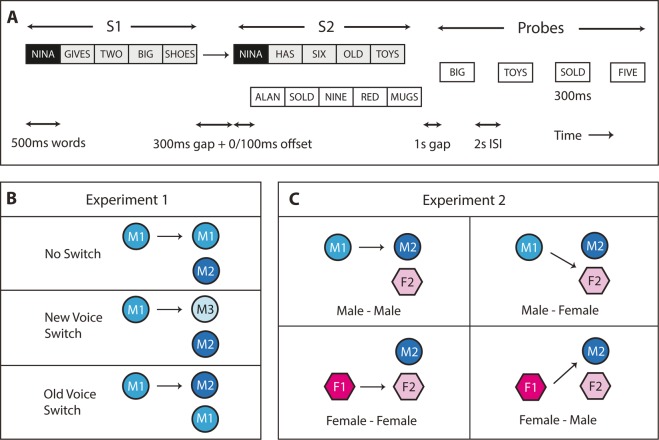
Experimental design and conditions. **(A)** Example of a single trial. Two target sentences (S1 & S2 in grey) with identical call-sign (black) were presented one after the other. S2 was presented with a masker sentence. Following this a series of 300 ms text probes containing one S1 target word, S2 target word, masker word, and fake word were flashed on a screen. **(B**,**C)** Voice conditions for Experiment 1 and 2 respectively. Voices are denoted by different colours, genders are denoted by different shapes (M = male, F = female), arrows denote allocation of the S2 target sentence.

Both experiments utilised the same sentence arrangement and task (Fig. 1A). Each trial began with a single target sentence presented in isolation followed by a silence gap, then a mixture of two overlapping sentences (one target and one masker). Subjects were instructed to listen to and remember all words in the first and second target sentences (S1 and S2) assigned with the same call-sign, while ignoring the non-target masker.

A 300 ms silence gap was introduced between phrases to approximate the average conversational turn-taking length in English speech^30^. This is also the estimated time it takes to switch top-down auditory attention^18,31^. The two sentences following the turn-taking gap were offset from each other by 100 ms to alleviate energetic masking and enhance grouping. Offset of target and masker sentences were counterbalanced between trials.

Stimuli were constructed using a custom MATLAB (Mathworks) script and played through a MOTU 24 I/O Audio Interface at 96 kHz sampling rate. Subjects listened to sentences diotically at 65 dB SPL using Sennheiser HD 280 Pro headphones in a sound attenuated room. This removed the influence of spatial cues and made the listening task exclusively non-spatial.

Four text probes were flashed one at a time on a computer screen at the end of each trial. Each set contained a word from: (i) S1, (ii) S2, (iii) the masker sentence, and (iv) a fake word not present in the trial. The order of these probes and the position of each in the sentence was selected using a randomised Latin square. Listeners had 2 seconds to press YES or NO using a button box to indicate if they heard the probe in either of the two target sentences. Responses and reaction times (for correct responses) were recorded for analysis.

### Experiment 1 – Cost of switching

There were three conditions in the first experiment: (i) no switch, (ii) new voice switch, and (iii) old voice switch (Fig. 1B). In the no switch condition, the voice remained the same between S1 and S2 (primed condition). In the new voice switch condition, the voice changed between target sentences with the S1 voice disappearing (unprimed condition). In the old voice switch condition, the voice changed between target sentences with the S1 voice becoming the masker (anti-prime condition). The two switch conditions were designed to look at the process of disengagement and re-engagement of endogenous attention with and without prior exposure to a voice.

There were 2 voices in each trial with a total of 3 male voices used interchangeably in the whole experiment. The three talkers had fundamental frequencies of 83, 96 and 101 Hz. The occurrence of all voices as target and masker were balanced. Subjects completed a short training set of 10 trials before completing a total of 96 trials (equal number of switch and no switch).

### Experiment 2 – Switching voices

There were 3 voices in each trial. A total of 2 male and 2 female voices were used in this experiment. The fundamental frequencies were 83 and 101 Hz for the male talkers and 192 and 216 Hz for the female talkers. All conditions involved a switch in voice. There were four conditions: (i) male to male, (ii) male to female, (iii) female to male, and (iv) female to female switch (Fig. 1C). The male and female voices after the switch were kept constant throughout the experiment to maintain an equivalent level of S2 masking. Subjects completed a total of 80 randomised trials (20 per condition).

### Data analysis

Hit rate for S1 and S2 probes and incorrect rejection rate for masker and fake word probes were calculated for each trial. A summed total score metric was calculated for each experimental condition. D prime was also calculated for both sentences using the following formulas: *d’*(S1) = *z*(fake word hits) − *z*(S1 target hits), *d’*(S2) = *z*(masker hits) − *z*(S2 target hits). Repeated measure one-way ANOVAs were performed to investigate differences between conditions with post-hoc pairwise comparisons performed using the Bonferroni correction.

### Reading span test

All subjects also completed a reading span test^32^ to assess working memory capacity. Subjects read a series of 3–6 sentences out aloud presented on a computer screen. After each sentence, subjects reported yes or no if the sentence made literal sense or not. After each set, subjects were required to list the first or last words of each sentence. The total number of words correctly recalled was used as an index of working memory capacity and correlated with listening task results.

## Results

### Experiment 1 – The cost of switching

**Figure 2:**
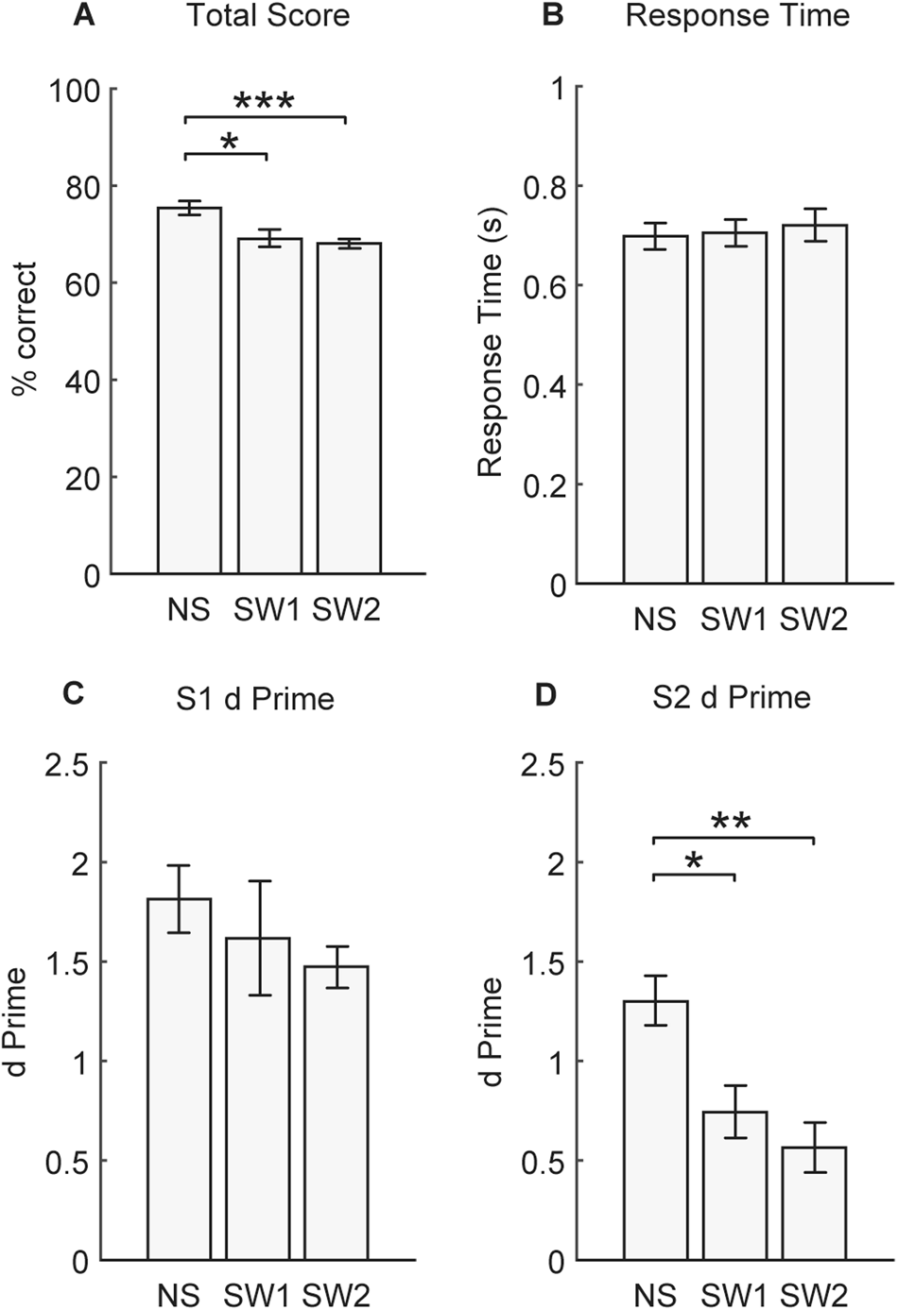
Experiment 1 results. **(A)** Total score, **(B)** Response Time, **(C)** S1 d prime, and **(D)** S2 d prime for no switch (NS), new voice switch (SW1), and old voice switch (SW2) conditions. Bars represent mean ± standard error of the mean (SEM), *p < 0.05, **p < 0.01, ***p < 0.001.

Figure 2a shows the mean total score for the three conditions in Experiment 1. The total score is a measure of correct performance across all four probes. A one way repeated measures ANOVA revealed a significant difference in total score between the conditions (F(1.4,19.6) = 10.2, *p* < 0.01). Performance was significantly higher in the no switch condition (mean = 75.8 ± 1.5%) compared to the new voice switch (mean = 69.1 ± 2.0%, *p* < 0.05) and old voice switch (mean = 68.1 ± 1.2%, *p* < 0.001) conditions. Despite this there were no significant differences in response times (Fig. 2b; F(2,28) = 1.7, *p* = 0.2).

A one way repeated measures ANOVA on S1 d prime (Fig. 2c) confirmed that there was no significant difference in target detection between conditions for words *prior to* the turn taking gap (F(1.4,19.5) = 1.12, *p* = 0.33).

However, there was a significant difference in target detection for S2 words *after* the turn taking gap (F(2,28) = 13.7, *p* < 0.001; Fig. 2d). S2 target sensitivity was significantly higher in the no switch condition (mean d’ = 1.3 ± 0.12) compared to both the new voice switch (mean d’ = 0.75 ± 0.13, *p* < 0.05) and old voice switch (mean d’ = 0.56 ± 0.12, *p* < 0.01) conditions.

**Figure 3:**
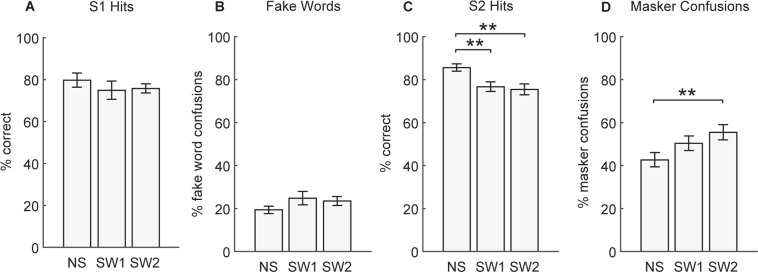
Experiment 1 results for each probe type. **(A)** S1 hits, **(B)** fake word confusions, **(C)** S2 hits, and **(D)** masker confusions for no switch (NS), new voice switch (SW1), and old voice switch (SW2) conditions. Bars represent mean ± SEM, **p < 0.01.

Figure 3 shows the performance broken down for each probe type. There were no significant differences in S1 hits (F(2,28) = 1.0, *p* = 0.37; Fig. 3a) or fake word confusions (F(2,28) = 1.73, *p* = 0.20; Fig. 3b) between conditions.

However, there were noticeable differences in performance for probes originating after the turn taking gap. A one way repeated measures ANOVA on S2 hits (Fig. 3c) confirmed a significant difference in S2 percent correct between conditions (F(2,28) = 8.9, *p* < 0.01). Post-Hoc tests revealed significantly higher S2 word detection for no switch (mean = 85.6 ± 1.8%) compared to new voice switch (mean = 76.7 ± 2.3%, *p* < 0.01), and for no switch compared to old voice switch (mean = 75.4 ± 2.6%, *p* < 0.01).

There were also significant differences in the number of masker confusions between conditions (F(2,28) = 6.8, *p* < 0.01; Fig. 3d). Post-Hoc tests revealed higher masker confusions in the old voice switch condition (mean = 55.5 ± 3.6%) compared to the no switch condition (mean = 42.6 ± 3.4%, *p* < 0.01).

**Figure 4:**
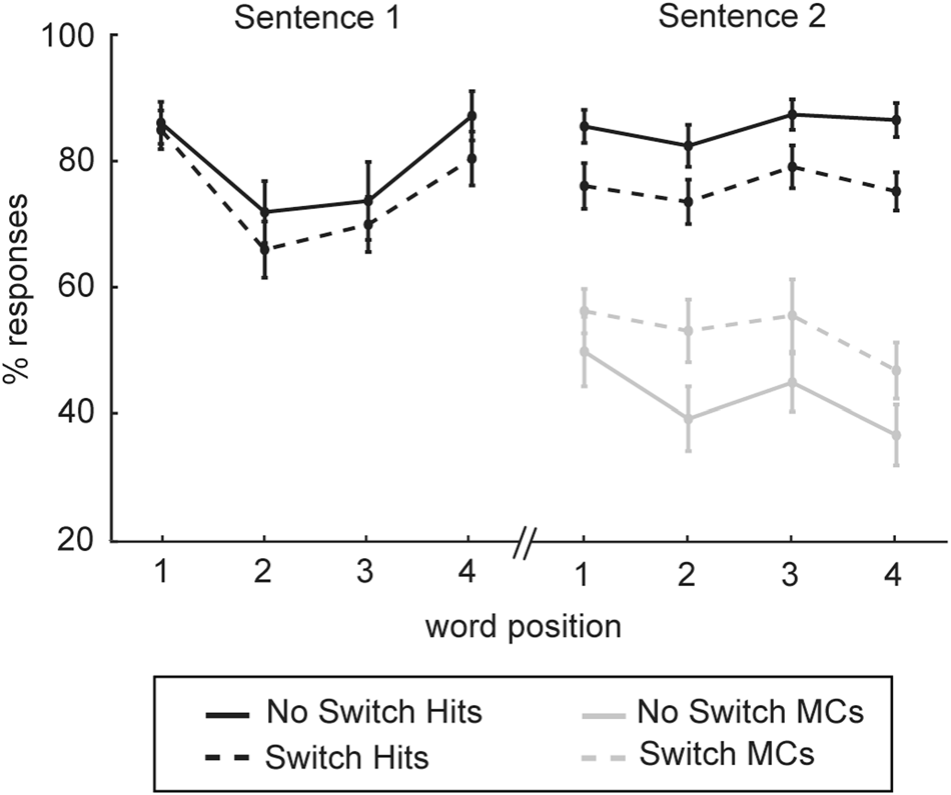
Experiment 1 data for each word position in a sentence. S1 and S2 hits (black) and masker confusions (grey) for no switch (solid) and switch (dashed) conditions. Data points show mean ± SEM.

Figure 4 shows the proportion of hits and masker confusion errors arranged by word position. Both switch conditions were combined for this analysis. For S1 hits, there was a significant effect of word position (F(3,42) = 8.4, *p* < 0.001) but no effect of switching. S2 hits were significantly higher in the no switch condition compared to the switch condition (F(1,14) = 16.9, *p* = 0.001), but there was no effect of word position. Masker confusions were significantly higher in the switch condition compared to the no switch condition (F(1,14) = 8.5 *p* < 0.05). The interaction between switching and word position was not significant for either S1, S2, or masker confusions.

Furthermore, there was no significant effect of probe position on recall and no interaction between probe position and condition. This indicates that performance across probes was equivalent with no temporal deterioration of accuracy during the recall phase.

### Experiment 2 – Voice differences

**Figure 5:**
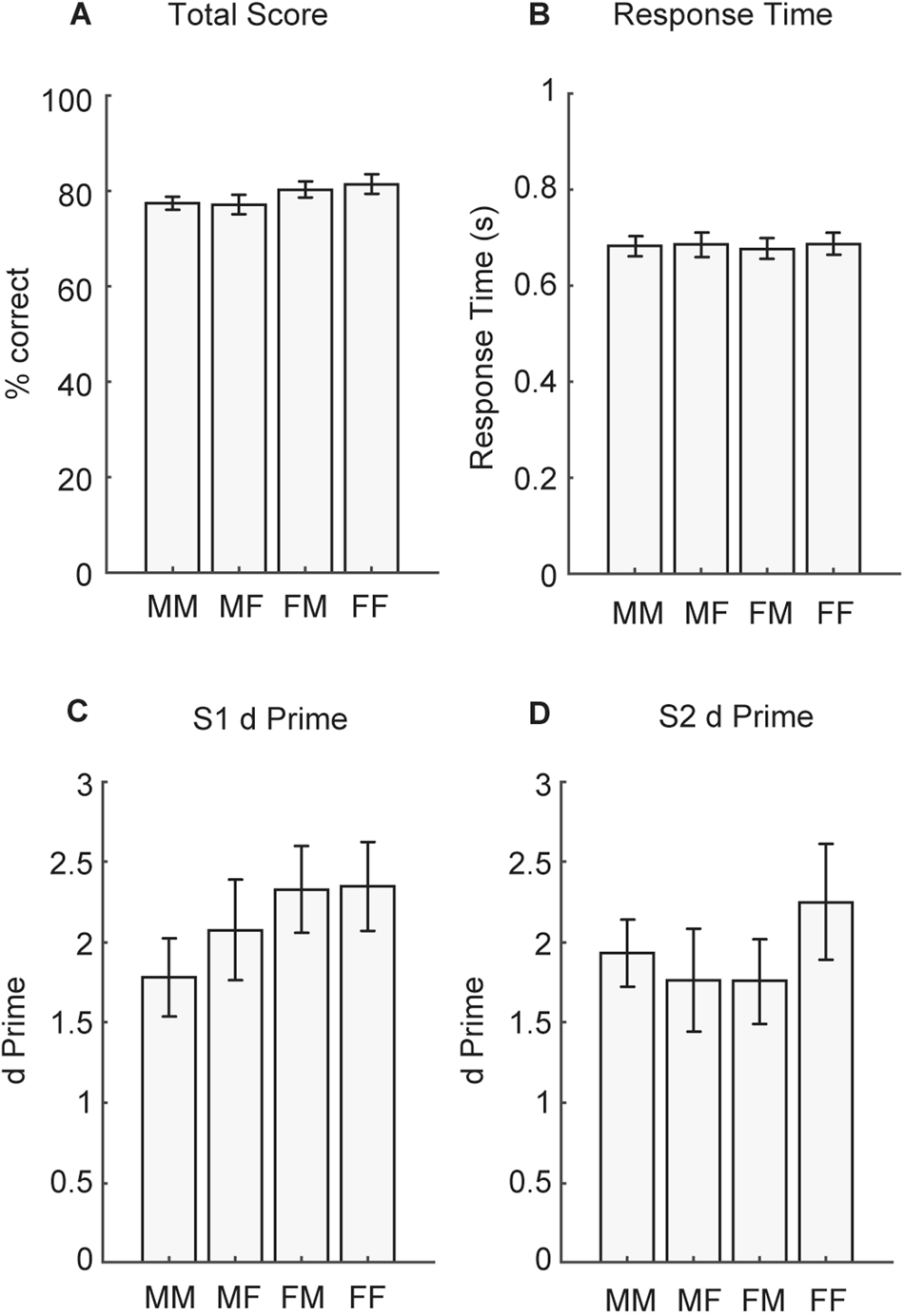
Experiment 2 results. (**A)** Total score, **(B)** Response Time, **(C)** S1 d prime, and **(D)** S2 d prime for male to male (MM), male to female (MF), female to male (FM), and female to female (FF) switch conditions. Bars represent mean ± SEM. All differences were not significant (*p* > 0.05).

Figure 5a shows the mean total score for the four conditions in Experiment 2. A one way repeated measures ANOVA revealed no significant differences in total score between the different voice switch conditions (F(3,42) = 2.2, *p* = 0.10). Like the results in the first experiment, there were also no significant differences in Experiment 2 response times between conditions (Fig. 5b; F(3,42) = 0.40, *p* = 0.75).

A one way repeated measures ANOVA on S1 data (Fig. 5c) confirmed that there was no significant difference in target detection between conditions *prior to* the turn-taking gap (F(2.0,27.9) = 1.2, *p* = 0.33). A one way repeated measures ANOVA on S2 data (Fig. 5d) confirmed that there was also no significant difference in target detection between conditions *after* the turn-taking gap (F(3,42) = 0.3, *p* = 0.73).

**Figure 6:**
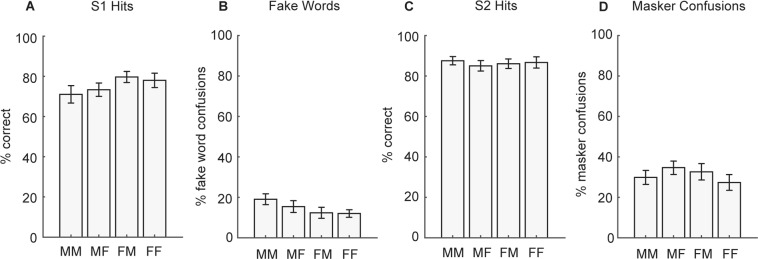
Experiment 2 results for each probe type. **(A)** S1 hits, **(B)** fake word confusions, **(C)** S2 hits, and **(D)** masker confusions for male to male (MM), male to female (MF), female to male (FM), and female to female (FF) switch conditions. Bars represent mean ± SEM. All differences were not significant (*p* > 0.05).

Figure 6 shows the mean performance for each probe type. There were no significant differences between conditions in S1 hits (Fig. 6a; F(3,42) = 203, *p* = 0.10), fake word confusions (Fig. 6b; F(2.1,29.6) = 2.7, *p* = 0.08), S2 hits (Fig. 6c; F(3,42) = 0.21, *p* = 0.89), or masker confusions (Fig. 6d; F(3,42) = 1.1, *p* = 0.38).

## Discussion

This study examined the process of switching non-spatial attention in a multi-talker conversation. Two experiments were conducted using matrix sentences to evaluate the recall of information following a single endogenous switch in attention. The first experiment established a perceptual cost in switching. Specifically, sentence recall was worse when a target voice switched compared to when it remained the same after a turn-taking gap. There was a decrease in target hit rate and sensitivity in both switch conditions. This highlights the cognitive demands of *re-engaging* attention on a new talker. There was also an increase in masker confusion errors in the old voice switch condition where the target became a masker. This demonstrates a significant cost of *disengaging* attention and shows that a voice can have a persistent biasing effect, even after it is no longer a target.

The second experiment investigated the effect of different talker combinations on switching performance. There was no change in performance when comparing within-gender and between-gender voice switches. This suggests that switching costs observed in this study were not influenced by the degree of differences in acoustic characteristics of the target talker.

This study investigated the acoustic and cognitive demands of non-spatial attention switching in the context of a conversation. Previous studies have examined the recall of only a limited number of items, usually digits or singular words, following a change in voice^21,22,24–26^. Here we probed the recall of two whole sentences (8 items) before and after a turn taking gap. This allowed for the examination of streaming and working memory demands over the course of an unfolding conversation.

The small but significant 7% decrement in total score between switch and no switch conditions in Experiment 1 is in line with previous studies which have shown switching costs of between 5–15% in normally-hearing listeners^9,13,15,18,22^. Here the degradation in S2 recall and sensitivity in both switch conditions indicates the cost is localised to after a turn-taking gap. This pattern of results supports the notion of a disruption to streaming following a change in target talker^9,22^. This study provides some insight into why this cost exists in a conversational setting. Here, we explore a number of factors including; (i) a cost re-engaging attention, (ii) a cost disengaging attention, and (iii) an increase in cognitive load resulting from the increased processing required by the switch.

Firstly, switching costs could be due to the process of re-engaging attention to a new target voice. Previous studies have shown that continuity in voice benefits selective attention^9,17,21,22^. A consistent voice provides the auditory system with an acoustic template, which assists in the extraction of a matching talker^21^. This explains why in the current study, performance was best in the no switch condition where a target voice remained the same between sentences. This also supports priming studies which have shown that intelligibility of a target word is best when it is preceded by a sentence spoken by the same talker^24–26^. However, unlike previous studies, where priming sentences were cues to voice repetition, the current paradigm shows the benefit extends to situations where voice repetitions are uncued. In our task, voice repetition trials were unpredictable and randomly mixed with switch trials. This is reflective of an ‘open conversation’ in the real world, where all talkers are free to contribute after a turn-taking gap.

The current study demonstrates the auditory system’s ability to use the previous voice characteristics in S1 to enhance detection of S2 words. This voice repetition benefit is both immediate and sustained across S2 word positions as seen in Fig. 4. Furthermore object attention builds up over time when the features of a scene are constant, adding to this perceptual benefit^9,22,33,34^. When there is an unexpected voice switch, this forces attention to be re-oriented which resets the process of object formation. Listeners cannot rely on the template of a previous voice and must adjust to new talker characteristics^21^. This re-initiation of streaming has a detrimental impact on encoding of a new target^9,22^, as seen with a decrease in S2 hits and S2 sensitivity in the current study. These results provide evidence for a cost of re-engaging attention after a change in talker.

Once exposed to a voice, listeners have a perceptual bias toward it^9,22^. This bias can improve performance if the target voice is preserved, but it can also hinder performance if it later becomes a masker. We observed this in the old voice switch condition in Experiment 1 where the original voice in S1 became a masker in S2. This condition was designed to assess whether there was an additional disengagement cost with switching. Listeners experienced a significant decrease in word recall and an increase in masker confusion errors in this condition. Here, the S1 target voice exerted a stimulus driven biasing of attention due to its previous importance. Even after a change in target, listeners continued to stream or attend to the original (now masker) voice well into the second sentence. This highlights the persistent bias an encoded voice can have in a conversation, and demonstrates the challenge dissociating from a previous target. These results here suggest that there is a significant component of disengagement effort associated with successfully switching attention.

In addition to dis-engaging and re-engaging attention, listeners are also faced with working memory demands in a conversation. Previous studies have shown that switching attention imparts a cognitive load which affects the encoding, storage and retrieval of auditory information^15,18^. This is because individuals have a finite working memory capacity which must be distributed across these multiple processes^35–37^. Switching requires effort^13,19^ which results in less resources devoted to processing of stimuli directly after a switch.

In Experiment 1, the drop in S2 hits following a voice switch is consistent with this hypothesis. Degradation in recall appears to be operating at a whole sentence level, affecting all S2 words equally (Fig. 4). This provides evidence that the cost is due to a global cognitive process rather than a word based streaming issue. If it was solely due to target re-orientation, we would expect an initial decline in S2 performance after a switch, followed by an improvement across words as object attention becomes more finely tuned^9,13^. However, we did not see an improvement across time, with no interaction between word position and switching performance. Furthermore, we observed a significant rise in masker confusion errors after a switch. This may reflect a rise in working memory demands which increases susceptibility to distractor interference^38–40^. Interestingly, recall of S1 words was minimally affected by a switch in attention (Fig. 4). This indicates that switching does not affect memory of words already encoded, it only adds to the difficulty of encoding future words.

Surprisingly, response times to probes were unaffected by experimental conditions. In contrast, previous studies have shown significant slower response times to switch compared to no switch trials^18,19^. The uniform response times observed here may be driven by the instruction given to subjects to respond as fast as possible to probes. This effectively constrained the amount of ‘thinking time’ or effort that subjects applied to their decision. Hence subjects traded off fast response times to the detriment of accuracy, with the cost being larger for switch conditions. This again supports cognitive spare capacity memory models^36,37^, showing that switching non-spatial attention adds to working memory load.

In contrast to our previous study^15^, we did not find a significant correlation between an individual’s working memory capacity and listening task performance. This is likely due to differences in experimental design. Even though both studies had a substantial working memory component, the current experiment is simpler in comparison with fewer masking sentences (1 vs 4) and a less demanding task (word recognition vs verbatim recall). Despite this, we were still able to observe a significant and comparable cost of switching. The current study emphasises the role of re-engagement, disengagement, and cognitive load in switching non-spatial attention. It is likely that a combination of these three factors contributed to the cost of switching observed here.

Experiment 2 examined the effect of voice differences in switching. There was no impact of target gender, fundamental frequency, or voice quality on all metrics of switching performance. This suggests that switching costs observed here are not primarily driven by differences in target talker characteristics. That is not to say that talkers cannot influence attention in a conversation. Voices can bias allocation of attention in a scene based on their intelligibility^27,28^ and their saliency^41^. The current study attempted to minimise the contribution of these two factors to see if there remained any differences in the cost of switching. Firstly, we used recordings from the Australian Matrix Sentence Corpus^20^ which contains words and talkers optimised and validated to be perceptually equivalent in noise. This allowed for the selection of talkers of similar intelligibility. Secondly, the design of the experiment also removed potential differences in masking by using the same pair of talkers after each switch gap. Furthermore, the closed set nature and the lack of contextual cues in the corpus removed any potential saliency in the content of the sentences. The counterbalancing of the occurrence of all talkers also ensured listeners were equally exposed to all voices.

After controlling for intelligibility and saliency, there did not appear to be differences in switching performance based on the assignment of target voice. This uniformity in switch costs despite voice differences implies that switching cost does not depend on talker characteristics. Voice differences are important in auditory scene analysis, in the initial separation and grouping of objects in a mixture^5,42^. But the degree of voice differences from one sentence to the next did not appear to affect the cost of switching. This suggests that costs may be binary in nature (same/different). After a turn-taking gap, the auditory system compares the voice in the new target sentence with the voice heard in the previous target sentence. If there is match in acoustic templates, there is no cost incurred^21^. However, if there is a mismatch, regardless of how large or small, there is a cost because of the need to disengage and re-engage attention. These data suggest that this comparison appears to be binary and may relate simply to whether the voices are judged to be the same or different and does not depend on the magnitude of differences in acoustic characteristics. This finding is consistent with our previous study on spatial switching which showed that the switching costs did not depend on angular size of the spatial shifts^15^. Together, this data supports the view that auditory attention is object based and that switching is operating as a binary comparison of two discrete entities.

In conclusion, this study has established that there is a significant perceptual cost associated with switching non-spatial attention in a conversational setting. Listeners must not only engage their attention on a new talker of interest but may also have to disengage attention from a previous target voice. This interruption to the streaming process introduces a cognitive load which impacts on the encoding and recall of a sentence directly after a switch. These costs appear to be unaffected by the degree of differences in target talker characteristics.

## Data Availability

The datasets generated during and/or analysed during the current study are available from the corresponding author on reasonable request.
